# Cold Atmospheric Pressure Plasma: A Growing Paradigm in Diabetic Wound Healing—Mechanism and Clinical Significance

**DOI:** 10.3390/ijms242316657

**Published:** 2023-11-23

**Authors:** Azadeh Barjasteh, Neha Kaushik, Eun Ha Choi, Nagendra Kumar Kaushik

**Affiliations:** 1 Department of Physics, Lorestan University, Khorramabad 68151-44316, Iran; barjasteh.a@lu.ac.ir; 2 Department of Biotechnology, College of Engineering, The University of Suwon, Hwaseong 18323, Republic of Korea; neha.bioplasma@gmail.com; 3 Department of Electrical and Biological Physics/Plasma, Bioscience Research Center, Kwangwoon University, Seoul 01897, Republic of Korea; ehchoi@kw.ac.kr

**Keywords:** cold atmospheric plasma, wound healing, diabetic wounds

## Abstract

Diabetes is one of the most significant causes of death all over the world. This illness, due to abnormal blood glucose levels, leads to impaired wound healing and, as a result, foot ulcers. These ulcers cannot heal quickly in diabetic patients and may finally result in amputation. In recent years, different research has been conducted to heal diabetic foot ulcers: one of them is using cold atmospheric pressure plasma. Nowadays, cold atmospheric pressure plasma is highly regarded in medicine because of its positive effects and lack of side effects. These conditions have caused plasma to be considered a promising technology in medicine and especially diabetic wound healing because studies show that it can heal chronic wounds that are resistant to standard treatments. The positive effects of plasma are due to different reactive species, UV radiation, and electromagnetic fields. This work reviews ongoing cold atmospheric pressure plasma improvements in diabetic wound healing. It shows that plasma can be a promising tool in treating chronic wounds, including ones resulting from diabetes.

## 1. Introduction

**Figure 1:**
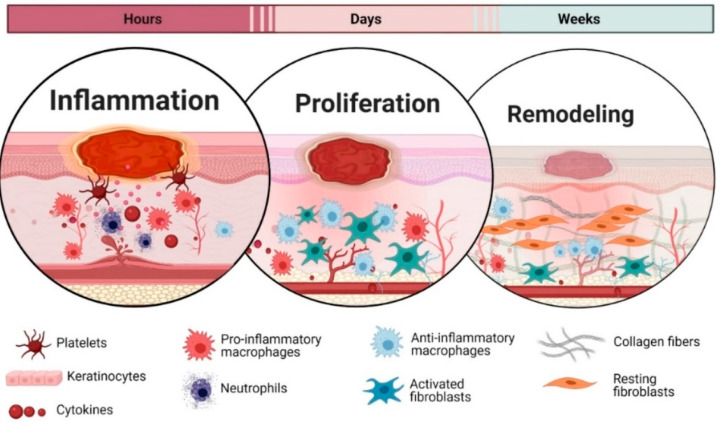
Schematic of wound healing phases [4].

Nowadays, one of the most common diseases is diabetes mellitus (DM); nearly 1.9 million new cases in the United States are diagnosed every year, and it is the ninth major cause of death all over the world. In diabetic patients, nearly 90 to 95% have type 2 DM [1]. The main risk factors for type 2 DM are genetics, obesity, and lifestyle [2]. Failure to control this type of diabetes leads to impaired wound healing due to abnormal blood glucose accumulation, and as a result, diabetic ulcers such as foot ulcers are formed. Wound healing is a complex process that is composed of four main mechanisms: hemostasis, inflammation, proliferation, and dermal remodeling, supported by continuous cellular events to efficiently repair damaged tissue and to quickly close the skin barrier [3]. This process is shown schematically in Figure 1. 

**Figure 2:**
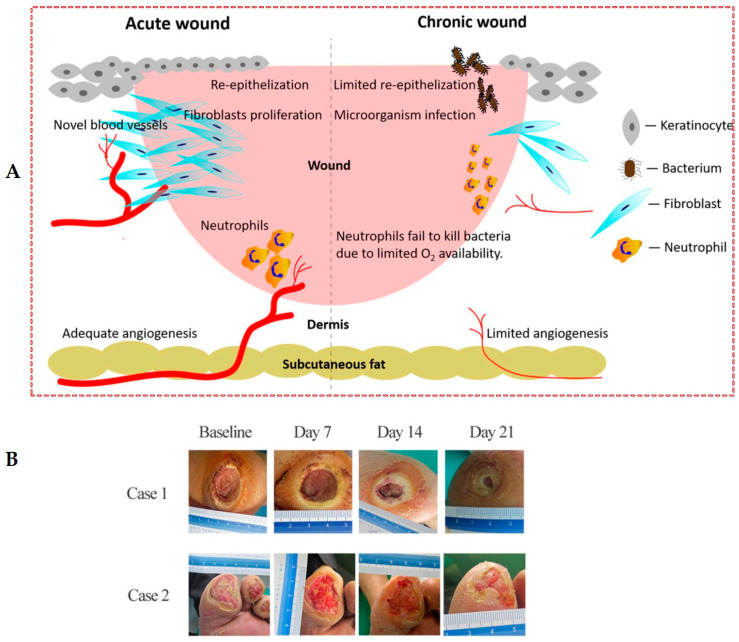
(**A**) The difference between chronic and normal wounds schematically [51]. (**B**) Effect of cold atmospheric plasma on diabetic foot ulcers [14].

Hemostasis is a mechanism in which immediately after injury blood clots are formed because of the rapid contracting of damaged blood vessels [5]. The inflammation phase is the primary defense against wounds that are started by injury-induced signals [6]. In the proliferation phase, keratinocytes, fibroblasts, macrophages, and endothelial cells are activated extensively to close wounds and start angiogenesis. Finally, dermal remodeling refers to restoring skin health from the dermis [7]. Whenever an injury occurs, the above-mentioned different mechanisms influence the healing process at the wound site. There are different factors such as obesity, diabetes, age, stress, and poor oxygenation that affect wound healing [8]. In diabetic patients, wound healing is a complicated mechanism and is associated with many factors, such as neuropathy, vascular diseases, and foot deformities [9], that cause wounds to fail to heal and are named chronic wounds because they do not heal for months or even years and often recur. Figure 2A schematically shows the differences between chronic and normal wounds. Cellular senescence is one of the main factors in impairing the healing of diabetic wounds [10]. Diabetic wounds have different characteristics such as infections and accumulating liquid and necrotic tissue, and proteinase and cytokines are increasingly released to disrupt the healing process. Due to poor blood supply to the wound site and neuropathy, the healing process is delayed, and increasing concentrations of interleukins and cytokines via the prolonged inflammatory phase cause delayed wound closure [11,12]. The standard treatment to cure diabetic foot ulcers includes glycemic control, using proper antibiotics to control infections, and locally includes intermittent rinsing and dressing. In most cases, these treatments are not enough to cure diabetic foot ulcers and lead to limb amputation [13]. Also, the costs for care of these wounds are significantly high while the survival rate in patients who need limb amputation is significantly low [13,14]. In diabetic patients, mitotic cells, which are responsible for injury repair, become senescent and non-proliferative. Therefore, their ulcers become refractory to healing and infection often causes them to be chronic [15]. A foot ulcer is the main chronic ulcer in diabetic patients that, in most cases, leads to limb amputation; nearly half of the amputation cases in different countries such as Romania, Germany, and Spain are due to diabetic foot ulcers [16]. In recent years, researchers have been trying to develop new treatment methods such as using nanomaterials to target drug delivery, gene delivery, tissue engineering, anti-bacterial activity, and using cold atmospheric plasma to significantly decrease the healthcare cost of patients and simultaneously alleviate patients’ pains [17,18]. It has been shown in in vitro and clinical research that cold atmospheric pressure plasma (CAP) can decrease the bacterial loads in diabetic foot ulcers and simultaneously accelerate wound healing without any side effects [1,19,20,21,22,23,24]. Plasma, named the fourth state of matter, is an ionized gas composed of electrons, ions, free radicals, excited species, UV radiations, gas atoms, molecules in a ground or excited state, and electromagnetic fields. The most significant characteristic of plasmas is their energy and electrical density. Plasmas based on their temperature are divided into two categories: nonequilibrium or cold plasma (because the electron temperature is high enough (10,000 K), while gas remains at room temperature) and equilibrium or hot plasma [25]. The use of cold plasma in the medical field dates back to the middle of the 19th century when Siemens, in the 1850s, wanted to clean contaminated water by a dielectric barrier discharge [26]. In the early years of the 20th century, cold plasma became very popular because of its low temperature, ease of working, lack of expensive equipment and vacuum pump, and ease of making. Since the antimicrobial effects of plasmas were proven in 1990, they have been widely used in medicine. Their promising applications include wound healing, dentistry, cancer treatment, ophthalmology, dermatology, surgery, and cosmetics [20,27,28,29,30,31]. This technology, which is used extensively in material processing, does not need expensive equipment and is easy to use. CAP is made in different forms, such as glow discharge or radio frequency discharges (RF), corona discharge, and dielectric barrier discharge (DBD) [32], of which the energy and electronic density are, respectively, 1–10 ev and ~10^10^ cm^−3^ [25]. Glow discharge or radiofrequency discharge is produced at low pressures when both electrodes are connected to a radio frequency voltage (0.04 to 13.56 MHz) or low frequency (50 Hz) [33]. Corona Discharge is formed when two electrodes with different sizes (one of them is pin-like) are connected to a low-frequency or pulsed high-voltage source. The plasma produced in this way is not homogenous and as a result, it is not desirable for material processing [25]. A DBD is made when at least a dielectric layer is inserted in the discharge gap between two electrodes: one of them is grounded and the other is connected to a high-frequency alternative current. Dielectric material prevents the generation of arc and, as a result, the gas temperature does not raise. DBDs have different geometries from surface DBDs to cylindrical ones; the latest is named CAP. CAPs are the innovative structures of DBDs; the main difference between them and surface DBDs is that in the first one, plasma is produced inside the dielectric tube and is then transferred to the ambient air by gas flow. At the same time, in the second one, plasma is produced in ambient air between two electrodes. Also, in CAPs, different gases can be used as feeding gases, while the surface DBD can be produced in ambient air [34,35,36]. In CAPs, plasma is ejected from a discharge tube like a plume, in which electrons have a high temperature ($\approx 10^{4}$ K) while ions and neutral species remain at room temperature. Also, charged particles in contact with air produce reactive oxygen and nitrogen species; some have short lifetimes and produce UV radiation. When these reactive species collide with the wound surface, the cascade of actions is produced, from physical effects such as generating reactive species, which are necessary for promoting wound healing, to biological effects such as cellular processes, which are responsible for DNA and bacteria cell damage. Therefore, by penetrating the microorganism’s cell membrane via making pores, these reactive species attack their DNA and inactivate them [37,38]. Also, these active particles, combined with UV radiation, stimulate skin regeneration via activating regeneration and cell migration [39]. As a result, they decrease wound infection, react with proteins, attack the lipids of tissue, decrease inflammation, prompt cell proliferation, and change the physical and chemical properties of the wound and, in this way, accelerate wound healing [1]. In recent years, the effect of CAPs on wound healing, especially diabetic wound healing, has been investigated extensively in clinical and case studies because they have shown a capacity to reduce bacterial load in wounds, promote rapid healing, faster tissue regeneration, and proliferation without any side effects, which is their novelty in comparison with other treatments [1,14,19,22,40,41,42,43,44]. kINPen, a pen-sized cold plasma jet, was the first plasma device used in 2013 as a medical device after going through preclinical and clinical trials. It is used for the treatment of acute, infected, and chronic wounds and skin diseases [45]. After kINPen, plasmaDerm VU-2010 and SteriPlas are other certified plasma tools used in clinical studies to treat acute and chronic wounds in humans [29,46,47]. They have shown that CAP can be an effective tool in diabetic wound healing, especially when used alongside standard care such as glycemic control, ordered rinsing and dressing, and using proper antibiotics. Rui et al. in 2020 [1] investigated the efficacy of cold atmospheric He plasma on Diabetic wound healing in vitro and in vivo. Their results showed that wound healing in diabetic mice that received plasma for 90 and 180 s was much faster in comparison with the control group. Also, their in vitro results showed that cold plasma significantly caused healing within 180 s of exposure time. Clinical trials of CAP treatment are limited to two different phases, I and II, because of the different mechanisms of action of the product. For example, in phase I, the anti-bacterial efficacy of CAP was examined in different studies to prove its non-harmfulness [47,48,49] and in phase II its safety was examined in different clinical trials for patients with various chronic wounds [46,50]. In 2020, Bernd et al. [44], in the first clinical trial, investigated the effect of CAPs on diabetic foot ulcers. In this clinical study, they assessed 45 patients with a mean age of 68.5 years and 65 diabetic foot ulcer wounds. In total, 33 wounds from 29 patients were randomly selected to receive CAP treatment and 32 wounds from 28 patients received placebo treatment. Four patients in the CAP group and three patients in the placebo group were active smokers. Their results showed that plasma can significantly reduce wound size and it does not have any side effects on patients. In another clinical work in 2020, Shahriar et al. [14] clinically investigated the effect of CAPs on the treatment of diabetic foot ulcers. For this study, they assessed 44 patients aged >18 years without any history of cancer, not pregnant or breastfeeding, divided into two groups, each with 22 patients. The first group received CAP treatment with He gas as feeding gas three times a week for 3 consecutive weeks in addition to standard care. Their results showed that cold plasma, three times a week for three consecutive weeks, effectively reduced bacterial loads and wound size compared to the control group. Figure 2B shows their investigation of the effect of cold plasma on two diabetic foot ulcers. This figure shows that plasma causes an improvement in wounds after 3 weeks. 

Some reports investigated the effect of different feed gases, such as He and Ar, on CAP in wound healing [52,53]. Their results showed that Ar promotes coagulation better, while He is more effective in healing. In all of the clinically mentioned works, the safety of cold plasma treatment on diabetic wound healing has been investigated too. They showed that CAP is a safe and painless treatment without any side effects. In this review, we investigate diabetic foot ulcers and the mechanism of their healing. In the following review, the effect of cold atmospheric plasma on different stages of diabetic wounds healing preclinically (in vitro and in vivo) and clinically will be investigated. In clinical studies, the results of clinical investigations of different patients with diabetic foot ulcers treated with cold atmospheric plasma are compared with standard care such as ordered rinsing and dressing, glycemic control, and using proper antibiotics.

## 2. Diabetic Foot Ulcers

When the wound healing process is prolonged for more than 30 days after injury or several months or never heals, the wound is named chronic [54]. All wounds can be chronic if they are not repaired through a typical sequence. The wound healing is dependent on the wound type and its severity. All wounds are divided into types, such as acute type due to surgery or injury, infectious type due to bacteria, viruses, or fungus, ischemic type due to weak blood supply, radiation type due to cancer therapy, and ulcerated type due to sores or ulcers. Each wound has a typical depth, location, and appearance [55]. Various factors can cause a wound to be chronic, such as advanced age, immobility, diabetes, smoking or using alcohol, nerve damage, radiation, traumatic injury, weak immune system, and malignancy. Chronic wounds have symptoms such as bleeding, swelling, fever, pain, itching, difficulty moving the injured area, and unpleasant odors. These wounds have persistent infections and form microbial biofilms that are resistant to drugs. These wounds also have senescent cells and reparative stimuli do not affect them [56]. Chronic wounds are classified into four groups: arterial, diabetic, pressure, and venous ulcers. Diabetic foot ulcers are the most common reason for limb amputation in recent years and form due to a combination of structural deformities, underlying neuropathy, and peripheral arterial diseases [16,57]. The foot deformity is caused by these combined effects, such as the inability to detect pain, dryness, and repetitive injury. Such deformities are usually created on toes or the plantar and have a crater-like appearance; tendons or the bone in deep structures are exposed [55]. They are deep or shallow; usually, a deep callus ring surrounds them.

## 3. Wound Healing Mechanism

Generally, wounds are divided into two types: acute and chronic. Acute wounds usually heal within two weeks. The healing process in chronic wounds is significantly longer because of persistent bacteria in the wounds. Wounds heal through four stages: hemostasis, inflammation, proliferation, and remodeling [58,59]. Chronic wounds also have such healing stages but with a delay because bacterial infection affects the healing process as they are refractory to tissue reconstruction. Immediately after injury, the hemostasis stage occurs to prevent bleeding. The blood vessels are constricted at this stage, and platelets are activated to stick together and aggregate at the injury site. Platelet activation is started when they are exposed to extravascular collagens. In contact with collagens, soluble mediators such as growth factors cyclic AMP (Cyclic adenosine monophosphate) and adhesive glycoproteins are released from platelets. After platelet accumulation, releasing clotting factors causes fibrin clot deposit at the injury site. Trapping platelets in the fibrin web makes clots bulky. Inactive clotting enzyme proteases will be activated on the surface of platelets’ membranes and clotting accelerates. The second stage of wound healing is inflammation, which starts within the first 24 h after injury. This stage of wound healing is significantly longer in chronic wounds. At this stage, the enzymes, such as histamine, which causes symptoms of inflammation such as redness, heat, swelling, and pain, fill granules released from mast cells. During the inflammatory stage, the main cells responsible for cleaning debris and infection from wounds and releasing soluble mediators such as growth factors and proinflammatory cytokines are neutrophils, monocytes, and macrophages. Neutrophils are the first inflammatory cells that respond to released mediators from platelets. These cells, responsible for phagocytizing and killing bacteria, are the body’s first defense against infection. Blood plasma exudation is increased at this stage, macrophages and granulocytes are recruited, necrotic tissue is degraded, and an antimicrobial environment is established [60]. As inflammatory cells extravasate into the wound, essential interactions between adhesion molecules and receptors are started. At first, adhered leukocytes to endothelial cell walls start decelerating and rolling on the surface of endothelial cells, and in this way, leukocytes are activated. Then, presented chemotactic signals outside the venule cause leukocytes to squeeze and migrate into wounded tissue. After neutrophils migrate into the wound site, they start the generation of oxygen-free radicals to kill phagocytized bacteria and remove components of the extracellular matrix by releasing high levels of proteases. After 2 to 3 days of injury, neutrophils are depleted by the apoptosis process, and tissue monocytes replace them. The third stage of wound healing is proliferation, in which the tissue structure is restored with the help of fibroblasts because fibroblasts respond to released soluble mediators by platelets and macrophages and migrate into the wound. Fibroblasts change their morphology after migration to the wound site to proliferate and synthesize collagen. This stage of healing is composed of some prominent events. Angiogenesis is the most crucial event in this stage, where new capillaries are replaced with damaged vessels and circulation is restored. Granulation and epithelialization are the subsequent essential events in this phase of wound healing. The last stage of wound healing is remodeling, in which the scar is replaced by granulated tissue because of the decreasing number of capillaries as they aggregate into larger vessels and increasing tissue tensile strength due to the reorganizing of collagen fibers [61]. Chronic wound healing is delayed due to various reasons:

- The infection of diabetic wounds is chronic and resistant to usual treatments due to limited reactive oxygen species (ROS) availability in these wounds because ROS are the main species that excite neutrophils to kill bacteria.
- There is inadequate angiogenesis in diabetic wounds because the keratinocytes and macrophages responsible for initiating angiogenesis are limited in diabetic wounds.
- Fibroblasts, responsible for wound granulation and contradiction of wounds, are limited in diabetic wounds.

As a result, finding a way to promote fibroblast, neutrophil, and keratinocyte production and angiogenesis without any side effects is essential to treating diabetic wound healing and preventing amputation. The following shows that CAP may be a promising way to treat these chronic wounds.

## 4. Wound Sterilization or Wound Disinfection by Cold Plasma

**Figure 3:**
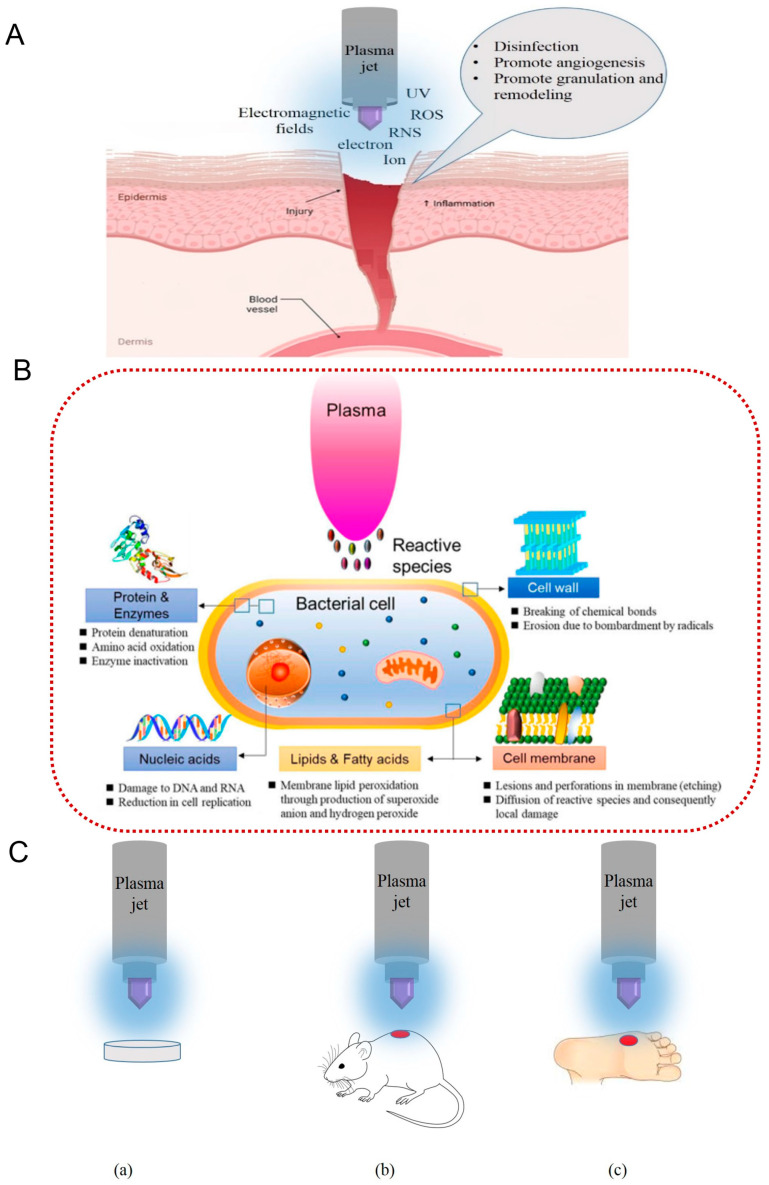
(**A**) different parameters of CAP that are effective in wound healing [73]. (**B**) effect of cold plasma on bacteria cells [20]. (**C**) different applications of CAP schematically. (a) effect of CAP in vitro, (b) effect of CAP in vivo, (c) effect of CAP clinically.

One of the main problems in treating chronic wounds is bacterial contamination because bacteria resist antimicrobial drugs and cause wounds to become established [21]. In contact with the wound, the first work of plasma is killing bacteria, which may cause the wound to become infected. Different studies have investigated the effect of CAP on bacteria killing and indicated that plasma can decrease the bacterial load in chronic wounds [62,63,64,65,66]. These reports show that plasma can be used in two ways to treat chronic wounds: directly and indirectly. In the direct method, plasma is generated in contact with the body surface in ambient air conditions [67,68,69]. In this way, the body surface acts as the second electrode. In an indirect method, plasma is first generated between two electrodes and is then transported to the wound site by the carrier gas [47,70,71]. Studies have shown that direct CAP treatment has higher antimicrobial efficacy than indirect CAP treatment in inactivating bacteria [47] because in these two treatments the concentration of charged particles is different; some reactive species that have a short lifetime are present in direct treatment while they are absent in indirect treatment [65]. In addition to the type of CAP devices, there are some factors such as the operating gas composition and flow rate, CAP exposure distance, CAP treatment time, and variation in source properties, such as frequency, voltage, and power, that affect the antimicrobial efficacy of CAPs [72]. Figure 3A shows the different parameters of CAP that are effective in wound healing, while Figure 3B summarizes the effect of CAP on the bacterial cell. In addition, Figure 3C shows different applications of CAPs in vitro (a), in vivo (b), and clinically (c).

As shown in Figure 2A, the plasma includes electrons, ions, ROS, RNS, UV radiations, and electromagnetic fields, which are effective in wound sterilization and, as a result, wound healing by inhibition of bacterial growth via the releasing of enzymes and the destruction of cell membranes by making pores in the walls of bacterial cells. Regarding operating gas composition, some studies have shown that adding gases such as O_2_ and N_2_ to the noble gases can increase the antimicrobial efficacy of CAPs by increasing reactive species [49,50]. Regarding gas flow rate, some reports indicate that by increasing the gas flow rate, the antibacterial efficacy of CAPs is decreased, while some reports state that it had the reverse outcome [72,74,75,76]. Thus, to have a successful CAP treatment, one should know the gas flow rate at which the most reactive species concentrate. Also, some reports have shown that the antimicrobial efficacy of CAP treatment is decreased by increasing the CAP exposure distance because the concentration of reactive species is reduced as a result of the short lifetime [64,77], meaning that some of them are annihilated before reaching the sample. The other factor that affects antimicrobial efficacy is the CAP treatment time. Research has shown that with increasing CAP exposure time, the antimicrobial efficacy of CAP increases due to more interaction between reactive CAP species and microorganisms [63,78]. The final parameter affecting CAP treatment’s antimicrobial efficacy is source parameters such as voltage amplitude, voltage frequency, and power supply, which should be optimized for better antimicrobial efficacy.

Research has shown that cold plasma, via three main mechanisms, such as electroporation and oxidation of cell membrane, intracellular oxidation and nitrification, and direct DNA damage, affects microorganisms, in which the first mechanism of making pores in cell walls causes the leakage of cell components. The second mechanism causes damage to the cell’s protein and disorder of gene expression, and the third one causes direct damage to the cell’s DNA [44,74,79]. In plasma, charged particles in contact with air produce reactive oxygen and nitrogen species (RONS) such as atomic oxygen (O), hydroxyl radicals (OH), singlet oxygen (O_2_), ozone (O_3_), hydrogen peroxide (H_2_O_2_), nitric oxide (NO), and nitrogen dioxide (NO_2_). Several studies have indicated that ROS have a primary role in deactivating microbes by damaging their membrane and losing viability [80]. In contrast, the other components of plasma, such as electric fields, UV radiation, and charged particles, maintain a small role [24,81,82,83]. During plasma treatment, these reactive oxygen species attack the microorganism’s cellular envelope and their intracellular components to deactivate them [62]. Reports have shown that between ROS, ozone is the main reactive species in killing bacteria via oxidizing the microorganism’s cell walls. Therefore, ozone increases cell membrane permeability and leads to cell death [62]. Another effective particle against the microbe’s cell membranes is hydroxyl radical. These species cause deterioration of the bacterial structure and, as a result, cell death by oxidizing the cell membrane [62]. In addition to the mentioned agents, UV radiation destroys the microorganism’s genetic material via photochemical reactions caused by photon absorption and by penetrating the cell wall, reacting with vital parts of the cell and, as a result, causing cell death [84]. UV radiation and charged particles accumulate on the cell wall, leading to cell membrane erosion and, as a result, cell death [85].

## 5. Investigation Studies of Plasma Effects on Diabetic Wounds In Vitro and In Vivo

Because diabetes is one main reasons for amputation all over the world, the issue of the treatment of diabetic wounds has been of interest in recent years. One of the main ways that has been investigated for treating diabetic wounds is cold plasma treatment because plasma has shown that it can decrease bacterial load and effectively heal wounds without causing any side effects. Generally, plasma is used in medicine as a direct and indirect treatment via activating liquids, such as water, which are called plasma-activated materials [26,86]. As previously shown in Figure 3C, the effect of cold plasma on diabetic wounds has been investigated in vitro, in vivo, and clinically, and different feeding gases such as He, Ar, and ambient air have been used to make cold plasma. Between them, helium cold plasma showed that it could reduce the PH of the wound site and, as a result, acidize wounds to decrease bacterial load and, by reducing inflammatory factors and stimulating cells to regenerate damaged tissue, promote wound healing [1,66,87]. The first effect of plasma, which is seen immediately after plasma treatment, is an antiseptic effect [14], in which the central role of antiseptic plasma is performed by ROS because these reactive species induce intracellular ROS in bacteria. Their accumulation causes an interruption in cellular function and, as a result, inactivation of bacteria [88]. These results also indicate that producing NO at a low concentration benefits the body, while a high concentration is harmful [89,90]. Thus, the plasma dose and treatment time is essential for wound treatment. To investigate the effect of treatment time on wounds, Rui He et al. [1] used helium cold plasma jets at different times, such as 90 and 180 s, in vitro and in vivo. In vivo, after 14 days of plasma treatment, they measured WBC, ALT, AST, BUN, and Cr levels and found no significant difference between the two groups. Their results suggest that plasma treatment does not have an essential effect on systemic inflammation or kidney and liver function. In vitro, they investigated human epidermal keratinocyte (HaCaT) cell migration after plasma treatment and found that plasma significantly accelerated HaCaT migration compared to the control. As a result, they concluded that 180 s treatment times in vitro and in vivo showed better results in wound healing. The other studies also emphasized that a low dose of plasma treatment or a shorter treatment time have stimulating effects by increasing proliferation and migration, and a high dose of plasma or a long treatment time have a lethal effect by inducing apoptosis, stopping proliferation, and causing cell cycle arrest [91,92]. Some other studies have investigated the effect of Argon cold plasma on wound healing. Their primary result was, at first, a reduction in wound surface area, reduction in infection symptoms, and reduction in microbial infection [44,52]. These studies showed that Argon cold plasma promotes coagulation, while helium cold plasma is better at healing [53]. In in vitro studies, the effect of cold plasma on the inactivation of bacteria, signaling events, and cell migration has been investigated [52,93]. In vivo studies have indicated that plasma not only has an antiseptic effect on the wound but it can also change growth factors, cause angiogenesis, cause cell migration and proliferation, and, in these ways, increase wound healing [23,42,94]. To investigate the effect of cold plasma clinically, for the first time in 2020, a group of researchers examined the effect of helium cold plasma on 44 patients with diabetic foot ulcers [23] in a randomized trial. They used plasma for 5 min thrice a week for three consecutive weeks. After three weeks, they observed that the wound size and bacterial load reduced significantly in plasma treatment patients. In another work [19], they investigated the clinical effect of cold plasma on inflammatory factors. For this study, plasma was used on 44 patients with diabetic foot ulcers. They observed that by using cold plasma on diabetic foot ulcers, cytokine levels decreased, inflammatory factors ameliorated, and, as a result, wound healing accelerated. Table 1 shows the clinical studies on the effect of CAP on diabetic foot ulcers. As a result, in vitro, in vivo, and clinical studies show that plasma treatment can be an effective tool for diabetic wound treatment without any side effects.

## 6. Growth Factors and Cytokine Changes and Immune Response after Plasma Treatment

**Figure 4:**
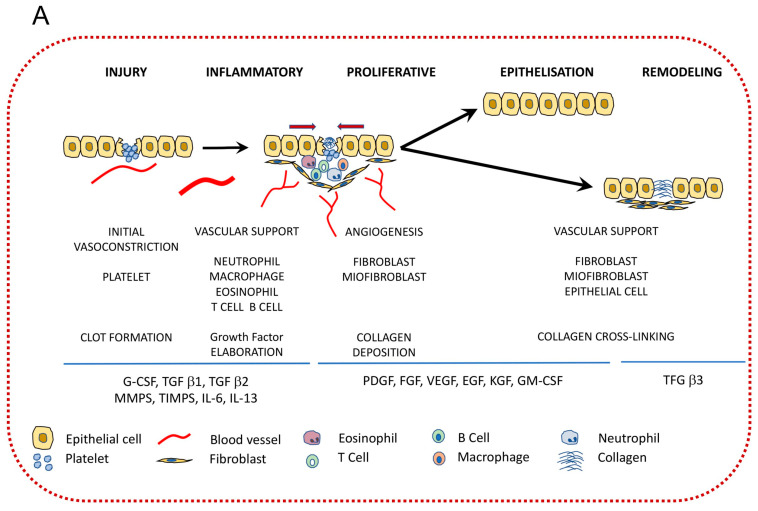

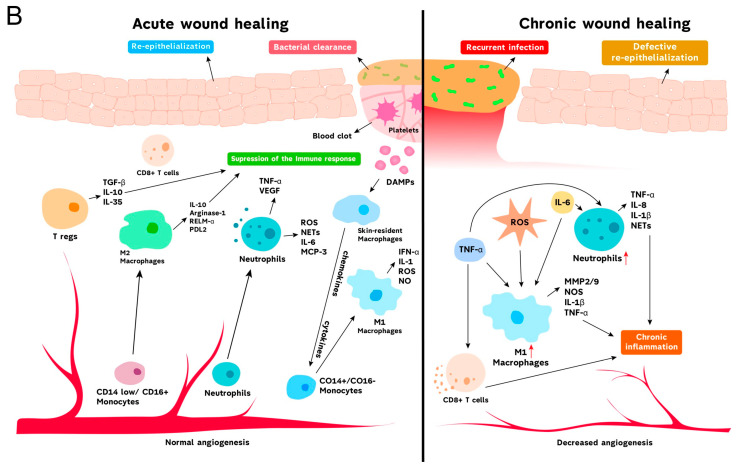
(**A**) growth factors and cytokines involved in wound healing [104]. (**B**) immune responses in acute and wound healing [105].

Wound healing is a process composed of overlapping some ordered events such as inflammation, proliferation, granulation tissue formation, and tissue remodeling. These processes are triggered and controlled by mediators that come from the wound site. When a wound is formed, the release of proinflammatory mediators causes inflammation. These mediators are molecular signals that cause inflammation by increasing inflammatory activities such as recruiting leukocytes and monocytes [98]. Cytokines are also small proteins that are secreted by cells and play an important role in interactions between different cells. They can affect the cells that secrete them (which is called the autocrine mechanism), the nearby cells (which is called the paracrine mechanism), and, finally, the distant cells (which is called the endocrine mechanism). Cytokines have different types. For example, when they are made by lymphocyte cells, they are called lymphokines; when they are made by leukocytes, they are called interleukins; when they are made by monocytes, they are called monokines; and finally, when they are made by chemotactic activities, they are called chemokines [99]. When a wound is created, growth factors such as EGF (Epidermal Growth Factor), PDGF (platelet-derived growth Factor), TGF β1 (Transforming Growth Factor Beta), and IL-1 (Interleukin) are released from platelets. These factors, which are polypeptide substances, are responsible for regulating growth, proliferation, cellular metabolism, and attraction of neutrophils to the wound site [100,101,102]. Neutrophils are predominant inflammatory cells that, by activation, can release enzymes such as elastase, protease, and collagens to remove damaged tissues at the wound site and clear the wound of infection [103]. Monocytes are differentiated into macrophages by TGF β1. The role of growth factors in wound healing is shown in Figure 4.

This figure shows that each mentioned growth factor does a different job. For example, EFG is a growth factor that stimulates cell growth, proliferation, and differentiation via binding to the EGFR (Epidermal Growth Factor response) [106]. PDGF incorporates other growth factors such as FGF-2, VEGF, and IL-8 by activating keratinocytes, and their migration across the cellular matrix plays an important role in the re-epithelialization process. Interleukins (IL) are important regulators of inflammatory processes produced by leukocytes that make cells turn on and off [107]. TGF β1 is responsible for enhancing angiogenesis via promoting endothelial cells and facilitating blood supply to the wound site [108]. The other growth factors, such as TNF-α (tumor necrosis factor) and IL-1α, are released from macrophages to initiate and regulate the proliferation of fibroblast and endothelial cells, in which fibroblasts are responsible for releasing collagen and other glycosaminoglycans to form the extracellular matrix, which is the main component of the granulation tissue [108]. IL-1α is a proinflammatory cytokine that plays an important role in the proliferation and migration of epidermal keratinocytes, prompting essential cellular events at an early stage of wound healing [100,109]. The other growth factors, such as BFGF (Basic Fibroblast Growth Factor) and VEGF (Vascular Endothelial Growth Factors), are released by endothelial cells, keratinocytes, and macrophages to initiate angiogenesis and collagen building for the growth of the granulation tissue and reduction in wound area and depth [43]. IL-8 is also a proinflammatory chemokine factor that, by secretion via autocrine activity, can promote cell proliferation, migration, and survival; as a result, increasing IL-8 ensures tissue remodeling [110], which decreases in diabetic wounds [111]. As a result, growth factors, via promoting endothelial and epithelial generation and stimulating angiogenesis, help collagen synthesis and, as a result, heal the wound. Indeed, a wound will be non-healing when there is a lack of these different growth factors [108]. Therefore, one of the ways to heal a chronic wound is by increasing these growth factors in patients. To increase these growth factors, researchers investigated the effect of CAP on the different growth factors in wound healing, and knowing them is important to treat chronic wounds, especially diabetic foot ulcers. For example, some reports have investigated the effect of CAP on interleukins and indicated that in the first treatment week of wound healing by CAP, IL-1α enhances and then merges into the phase of IL-8 values, meaning that IL-1α induces IL-8, which ensures better healing of diabetic wounds [19,52,112]. As a result, using CAP causes rebound phenomena that at first promote fibroblast migration to the wound site and then promote the synthesis of new granulation tissue [43,113,114]. These reports have also shown that by using CAP treatment in the first week, TNFα, which is responsible for the presence of macrophages and neutrophils, increases. This result is because ROS in cold plasma, by attacking the low-density lipoprotein particles (LDL), cause oxidation of lipids and proteins, which are the main components of LDL particles. Receptors then uptake these oxidized LDL species, such as those that are on the endothelial cells and macrophages, and, as a result, increase the release of inflammatory cytokines such as IL-1 and TNFα, which increases these proinflammatory factors and causes the wound to overcome its chronic state and to enter into the healing phase [115]. Some reports showed that FGF-2 and VEGF-A are significantly increased during plasma treatment because reactive oxygen species and reactive nitrogen increase the synthesis of these factors [116], which in turn have a beneficial effect on wound healing via the paracrine mechanism by enhancing vascularization, angiogenesis, proliferation, induction of granulation, and epithelialization. By increasing fibroblasts during CAP treatment, growth factors such as CTGF (connective tissue growth factor) and Cyr61, which are secreted from fibroblasts and are responsible for cell migration to the wound site, are also increased [117,118]. All of these processes are due to nitric oxide generation because CAP treatment increases the enzymes that are responsible for synthesizing nitric oxide, endothelial NO, and pro-angiogenic markers such as PDGFβ [43,95,119,120,121]. It has been proven that nitric oxide production in CAPs can promote cell migration and endothelial cells, which are useful for vascularization [122]. In other words, these factors, along with IL-8, PDGF, and TGF β11, by activating keratinocytes and their migration and proliferation across the extracellular matrix, play an important role in re-epithelialization and covering the wound site. Reports have indicated that PDGF and its receptor are decreased in diabetic mice, while CAP can increase them via hydroxyl radicals which are present in cold plasma [122].

## 7. Angiogenesis

The physiological process in which new blood vessels grow from the existing vessels is called angiogenesis. It is an essential process during wound healing [123]. Angiogenesis should be promoted to improve wound healing. Different factors such as growth factor VEGF, fibroblast growth factors (FGF), cytokines, reactive oxygen species (ROS), and nitric oxide (NO) can stimulate angiogenesis [91]. In diabetic patients, insufficient vascularization is one of the main reasons for impairing wound healing [124]. It is expected that CAPs, by the production of ROS and nitric oxide, have a positive effect on angiogenesis. CAPs can affect angiogenesis in two ways: directly and indirectly. Presently, some crucial factors of plasma called reactive oxygen species (ROS) and reactive nitrogen species (RNS), such as hydroxyl radicals (OH), hyper oxides (O_2_^−^), hydrogen peroxides (H_2_O_2_), nitric oxide (NO), proxy nitrites (ONOO^−^), and nitrogen oxides (NO_2_), can stimulate vascularization through the angiogenic growth factor mechanism [95,125]. Some of these reactive nitrogen species, such as nitric oxide, which is produced by neutrophils and macrophages during the inflammatory phase [126], form blood vessels in the wound area and perfuse blood into the wound site [127].

Therefore, CAPs can promote the angiogenic process by changing nitric oxide in the wound site [122]. Reactive oxygen species can also regulate the formation of blood vessels at the wound site and, in this way, perfuse blood into the wound healing area [127]. For example, some reports have indicated that hydroxyl radicals and hydrogen peroxides are responsible for enhancing tube formation [120,128]. Then, by using plasma, we can manipulate these reactive species numbers at the wound site. Also, studies have indicated that hydroxyl radicals and hydrogen peroxide may be responsible for enhancing tube formation by using endothelial cells [128]. In addition, plasma can increase the enzymes accountable for catalyzing nitric oxide synthase. Studies indicate that not only the reactive species type but also their concentration is an essential factor in wound healing. For example, a low nitric oxide concentration is necessary for the growth and inhibition of apoptosis, while a high concentration causes cell cycle arrest or apoptosis [129]. This result indicates that the dose of plasma or treatment time should be considered for wound healing because a low dose or short treatment time benefits wound healing and a high dose or long treatment time is destructive. Also, through the release of pro-angiogenic factors such as fibroblast growth factor 2 (FGF-2), cold plasma directly affects endothelial cell proliferation [125,130]. These cells stimulate fibroblasts to form granulated tissue and reduce wound area and depth [110]. In addition to FGF-2, vascular endothelial growth factors (VEGF) are another critical factor in angiogenic growth because these factors, by regulation of vascular permeability and angiogenesis, induce collagen building and epithelialization and, as a result, wound healing. Both of these factors cooperate in the activation of keratinocytes and, by influencing their migration to the wound site, cause them to proliferate to re-epithelialize and, as a result, cover the wound surface [100]. Reports show that CAPs, by their reactive species and UV radiations, can increase growth factors such as FGF-2 and VEGF in diabetic wounds and in this way help angiogenesis and, as a result, wound healing [1,19,43,95]. Not only reactive species cause angiogenesis but also electromagnetic fields along with plasma may cause angiogenesis because reports show that electromagnetic fields cause modulation of the movement of immune cells such as granulocytes and macrophages and the migration of skin cells such as keratinocytes; as a result, they can modulate fibroblast proliferation and cause angiogenesis [131,132,133]. CAPs also indirectly build relations between keratinocytes and fibroblasts and endothelial cells, called the paracrine mechanism, which promote angiogenesis-related factors in endothelial cells [43]. This result indicates that plasma can indirectly activate endothelial cells. Reports also show that CAP treatment induces different expression profiles and different kinds of factors between fibroblasts, keratinocytes, and endothelial cells. Therefore, the induction and amount of angiogenesis-related factors are dependent on the cell type’s sensitivity to the CAP [95].

## 8. Macrophages Changes after Plasma Treatment

**Figure 5:**
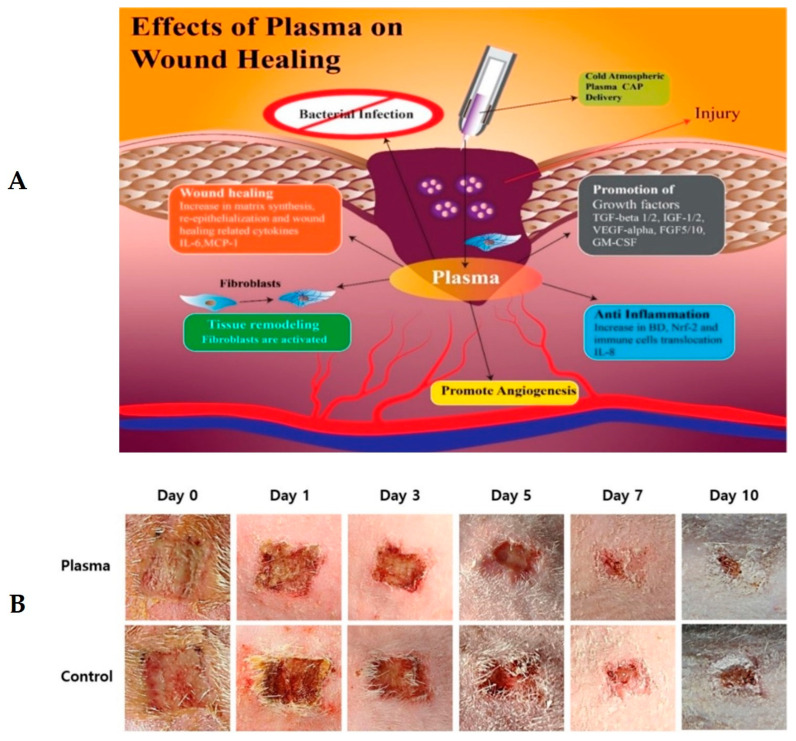

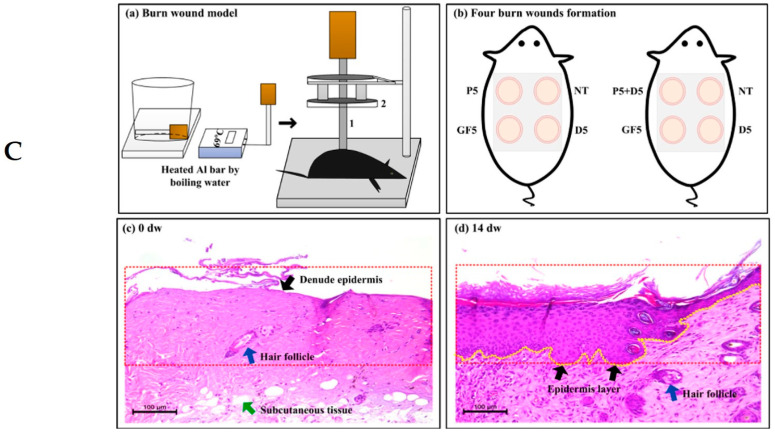
(**A**) CAP application on the wound increases growth factors and promotes macrophage recruitment for angiogenesis [20]. (**B**) representative gross photos of wound healing and closure in the plasma-treated and control groups by time [40]. (**C**) micro-plasma system effect on burn wounds in mice [139].

Macrophages are potent immune cells extensively used in the body to maintain hemostasis and resistance against invasive pathogens. These cells have a fundamental role in wound healing by their polarization [134,135] and are also critical elements in the inflammation phase. Generally, there are two types of polarized macrophages: M1, or classically activated macrophage, and M2, or activated macrophage [136], which play an essential role in repairing wounds. In normal wounds, M1 macrophages convert to M2 ones, but this process is impaired in diabetic wounds due to angiogenesis and collagen decreasing during wound closure [137]. M1 and M2 macrophages have two distinguishing features: functional plasticity and diversity [107]. M1 macrophages are responsible for enhancing the ability of antigen presentation, phagocytosis, and proinflammatory factors, while M2 macrophages are responsible for decreasing inflammatory response and increasing angiogenesis and tissue repair. They also degrade necrotic tissue by secreting extracellular enzymes and, as a result, resolve inflammation. In diabetic wounds, M1 macrophages are excessive, but M2 macrophages are inadequate in the proliferative stage. This process, which causes impaired diabetic wound healing, should be reversed to enhance wound healing. In 2019, Donghai et al. showed that a helium plasma jet can regulate the secretion of macrophages to limit inflammatory response [22]. In 2020, Mohammad Reza et al. showed that using cold atmospheric pressure plasma can reverse this process [19]. It means that plasma can decrease inflammatory factors and, by increasing angiogenesis factors, cause rapid wound healing in patients with chronic diabetic wounds. In 2021, Cong et al. [138] investigated the effect of cold atmospheric Argon plasma on macrophage changes in burn wounds. They showed that cold plasma induces M2 macrophages or anti-inflammatory macrophages by its reactive oxygen species and increases fibroblast migration, which are the leading causes of wound healing promotion. One year later, Jonas et al. investigated the effect of cold atmospheric plasma on enhancing growth factors in chronic diabetic wounds [43]. Their results showed that plasma, by activating macrophages responsible for inflammatory response, causes wounds to overcome their chronic states [43]. Figure 5A schematically shows the effect of CAP on increasing growth factors and promoting macrophage requirements, which are necessary processes for angiogenesis. Also, Figure 5B shows the effect of CAP on wound healing and wound closure in vivo. This figure shows that the plasma-treated diabetic wound, compared with the control wound, after day 5 presents more accelerated wound closure. To assess the effect of cold atmospheric plasma on burn wound healing via the stimulation of angiogenesis and epithelialization processes, Ngo et al. used Ar/N2 micro-plasma, which is shown in Figure 5C. After making four wounds on each mouse with an Al solid bar (Figure 5C(a,b)) and using different treatments such as plasma exposure, no plasma exposure, and only gas exposure, covered by an occlusion, they used hematoxylin and eosin staining, which is shown in Figure 5C(c,d). Their investigations showed that plasma, via two main mechanisms, can help wound contraction. At first, plasma reactive species such as ROS and RNS, through enhancement of the inflammation phase, stimulate the epithelialization process and then continuously stimulate the proliferation phase.

## 9. Insulin and Enzyme Activity Changes after Plasma Treatment

Insulin is a hormone secreted from B cells of pancreatic islets of Langerhans. It is responsible for facilitating glucose uptake, regulating carbohydrate, lipid, and protein metabolism, and increasing cell division and growth to control the level of blood glucose [140]. When there is not enough insulin secretion or there is no response to insulin, diabetes, which is a chronic metabolic disorder, occurs [141]. In recent years, different researchers have investigated the effect of cold plasma on insulin changes and enzyme activity. They have shown that reactive oxygen and nitrogen species have a two-fold nature in diabetes, meaning that while these species are necessary to the performance of insulin, they can also cause resistance of the body’s cells to insulin via induction of oxidative stress, depending on plasma properties such as plasma source type, feeding gas, amplitude and frequency of applied voltage, and espouser time [126,142]. Oxidative stress is a state in which antioxidants are increased, and as a result, the balance between their production and storage is lost. The oxidative stress changes the insulin signaling molecules’ structures or their pathways because they belong to the free radical species group that is highly reactive and, by attacking the cellular structure, causes oxidative damage to the cell or cell death [115,126]. Therefore, decreasing oxidative stress leads to a decreased resistance of the body and cells to insulin and, as a result, improvement of insulin performance. Recent studies have investigated the effect of plasma on oxidative stress and blood glucose levels (BGL) in in vivo and in vitro treatments [1]. For this investigation, H_2_O_2_ levels in the blood were measured. These studies have shown that in vitro treatment with cold plasma, both long-lived (such as ozone (O_3_), Hydrogen peroxide (H_2_O_2_), nitrite (NO_2_^−^), and nitrate ions (NO_3_^−^)) and short-lived species (such as Hydroxyl (OH), superoxide (O_2_^−^), and singlet oxygen (O)) affect Glutathione peroxidase (GPx).

In contrast, in vivo treatment of long-lived species affects GPx; GPx is an enzyme activity that converts H_2_O_2_ into water in mitochondria and cytosol. Plasma increases antioxidant activity because plasma treatment leads to increasing GPx and, as a result, causes an increase in H_2_O_2_ decomposition and, as a result, reduces oxidative stress and improves insulin performance [143]. Therefore, plasma can reduce BGL and has a positive effect of reducing AGEs (advanced glycation end-products) because the reduction in BGL leads to a reduction in the non-enzymatic glycation process of lipids or proteins due to the reduced exposure to glucose [144]. AGEs are produced when carbonyl groups in carbohydrates, like glucose, bond without enzyme intervention with free amino groups in a biomolecule, and their formation occurs along with the formation of oxidative stress [145,146]. Therefore, reducing AGE formation leads to lowered oxidative stress formation and, as a result, improved insulin performance. Different studies have investigated the effect of radiation on glucose metabolism [147,148,149]. Their results suggest that plasma radiations may also be effective in the promotion of glucose metabolism and, as a result, reduce BGL.

## 10. Conclusions

This review summarized studies on the effect of cold atmospheric pressure plasma on diabetic wound healing. The effect of cold plasma on different steps of wound healing has been extensively investigated. These studies have shown that cold plasma can disinfect wounds with its reactive species in the first step. This step is one of the main steps in wound healing because the bacteria in chronic wounds is resistant to traditional treatments, such as drugs used to treat infection. Then, these reactive species extensively participate in processes such as modulating inflammation, the secretion of growth factors, angiogenesis, migration, and tissue proliferation. Studies investigating the effect of CAP on diabetic wound healing have been done in vitro, in vivo, and clinically. All of these studies have indicated that plasma in moderate doses can heal chronic wounds, such as diabetic ones, by decreasing the bacterial load, reducing the wound surface, and reducing the time to wound closure. As a result, CAPs in the future will be a promising tool to treat diabetic wounds and, in this way, can prevent limb amputation in diabetic patients. Still, there are some questions that should be answered before using this technology extensively in diabetic wound treatment, such as what is the appropriate time for treatment and which characteristics of a wound have the best response to CAP.

## Figures and Tables

**Table 1:** Clinical studies on the effect of CAP on diabetic wound healing.

Reference	Method and Results	Year
[95]	Twenty-nine patients received CAP in argon gas once daily for five consecutive days and then three treatments every second day (8 times within 14 days). The wound surface area, clinical signs and symptoms of infections, and microbial infection were reduced significantly in comparison with the control group.	2018
[41]	Fouty-five patients received CAP in argon gas with eight plasma treatments in 14 days. Wound surface, clinical infection, microbial load, and the time for wound reduction decreased significantly in comparison with the control group.	2020
[19]	Twenty-two patients received CAP for 5 min three times a week for 3 consecutive weeks. Bacterial load and inflammatory factors decreased, angiogenesis increased, and wound healing accelerated.	2020
[96]	Ten patients received CAP in helium gas twice a week for six consecutive weeks. Wound size and bacterial load were reduced significantly compared with the control group.	2021
[97]	Ten patients received CAP in argon gas three times in the first week, twice in the second week, and once in the third week. The wound area and wound pH, and, as a result, the wound infection, were reduced significantly in comparison with the control group.	2022

## Data Availability

Not applicable.
